# A Mathematical Model for Enzyme Clustering in Glucose Metabolism

**DOI:** 10.1038/s41598-018-20348-7

**Published:** 2018-02-09

**Authors:** Miji Jeon, Hye-Won Kang, Songon An

**Affiliations:** 1 Department of Chemistry and Biochemistry, University of Maryland Baltimore County (UMBC), 1000 Hilltop Circle, Baltimore, MD 21250 USA; 2 Department of Mathematics and Statistics, University of Maryland Baltimore County (UMBC), 1000 Hilltop Circle, Baltimore, MD 21250 USA

## Abstract

We have recently demonstrated that the rate-limiting enzymes in human glucose metabolism organize into cytoplasmic clusters to form a multienzyme complex, the glucosome, in at least three different sizes. Quantitative high-content imaging data support a hypothesis that the glucosome clusters regulate the direction of glucose flux between energy metabolism and building block biosynthesis in a cluster size-dependent manner. However, direct measurement of their functional contributions to cellular metabolism at subcellular levels has remained challenging. In this work, we develop a mathematical model using a system of ordinary differential equations, in which the association of the rate-limiting enzymes into multienzyme complexes is included as an essential element. We then demonstrate that our mathematical model provides a quantitative principle to simulate glucose flux at both subcellular and population levels in human cancer cells. Lastly, we use the model to simulate 2-deoxyglucose-mediated alteration of glucose flux in a population level based on subcellular high-content imaging data. Collectively, we introduce a new mathematical model for human glucose metabolism, which promotes our understanding of functional roles of differently sized multienzyme complexes in both single-cell and population levels.

## Introduction

Glucose metabolism consists of glycolysis and gluconeogenesis. Glycolysis converts glucose into pyruvate, whereas gluconeogenesis reverses the sequential reactions to produce glucose^1^. In normal healthy cells, glucose is converted into pyruvate. Pyruvate then shuttles to mitochondria for oxidative phosphorylation in the presence of oxygen. If the oxygen is limited, however, pyruvate becomes lactate. In addition to its association with energy metabolism, glucose also plays an important role in building block biosynthesis, including the pentose phosphate pathway and serine biosynthesis. This is particularly important in cancer cells because glucose metabolism is dysregulated in proliferating cancer cells in which glucose diverts into building block biosynthesis even in the presence of oxygen. Hence, understanding the mechanism of glucose flux regulation between energy metabolism and building block biosynthesis is important to address altered metabolic phenotypes in human cancer as well as other chronic diseases, including but not limited to diabetes and obesity^2^.

Meanwhile, multienzyme metabolic complexes catalyzing glycolysis have been identified in various organisms, including protists, plants, yeast, mammalian neurons and erythrocytes, and human cancer cells^3^. Recently, it has been shown that human liver-type phosphofructokinase 1 (PFKL) forms cytoplasmic clusters in human cancer cells and further colocalizes with other cytoplasmic rate-limiting enzymes of the pathway, including human liver-type fructose-1,6-bisphosphatase (FBPase), pyruvate kinase M2 (PKM2), and phosphoenolpyruvate carboxykinase 1 (PEPCK1), thus indicating the formation of a multienzyme complex, namely the glucosome^4^. The size of glucosome clusters becomes larger in human breast carcinoma cells (Hs578T), relative to non-cancerous human breast tissue cells (Hs578Bst), demonstrating the spatial alteration of glucose metabolism in cancer cells^4^. Similarly, tumor-promoting hypoxic conditions also increased the size of glucosome clusters in human hepatocellular carcinoma cells (HepG2)^5^. Importantly, our quantitative high-content imaging data^4^ supported a hypothesis that the glucosome clusters regulate the direction of glucose flux between energy metabolism and building block biosynthesis in a cluster size-dependent manner. Unfortunately, however, it has been challenging to prove the hypothesis experimentally by measuring metabolic activities of various sized glucosome clusters at subcellular levels.

In parallel, several mathematical models for glycolysis have been developed using ordinary differential equations (ODEs) for various model organisms: e.g., bacteria, yeast, virus and human (erythrocytes and liver cells)^6–11^. Along with incorporating *in vitro* kinetic parameters of the enzymes in glycolysis, some ODE-mediated models describe the importance of bistable behavior in glycolysis with strong emphasis on the known allosteric regulation of the rate-limiting enzymes^12^. Particularly, recent mathematical models for human liver or cancer cells cover a complicated network beyond glycolysis by including the pentose phosphate pathway, serine biosynthesis, glutamine metabolism, and/or mitochondrial metabolism^13,14^. However, mathematical models for glucose metabolism have barely explained yet how a multienzyme complex influences glucose flux in cancer cells in a cluster size-dependent manner.

In this work, we have constructed a mathematical model to understand how the formation of a multienzyme complex in glucose metabolism affects glucose flux in cancer cells. Since the glucosome is composed of at least four cytoplasmic rate-limiting enzymes of glucose metabolism^4^, we have formulated a model to accommodate all the members of the glucosome. Our model indeed reveals the impact of their spatial complexation *in silico* on temporal changes of glycolytic metabolites at a subcellular level in single cells. We further show that our model is capable of predicting metabolic outcomes of a population of cells based on the relative distribution of glucosome clusters in the population. In conclusion, our mathematical model includes not only enzyme kinetics and their allosteric regulation but also spatial compartmentalization of the rate-limiting enzymes of the pathway, thus allowing us to understand the metabolic contributions of glucose flux at both single-cell and population levels.

## Model and Methods

### A Mathematical Model

**Figure 1:**
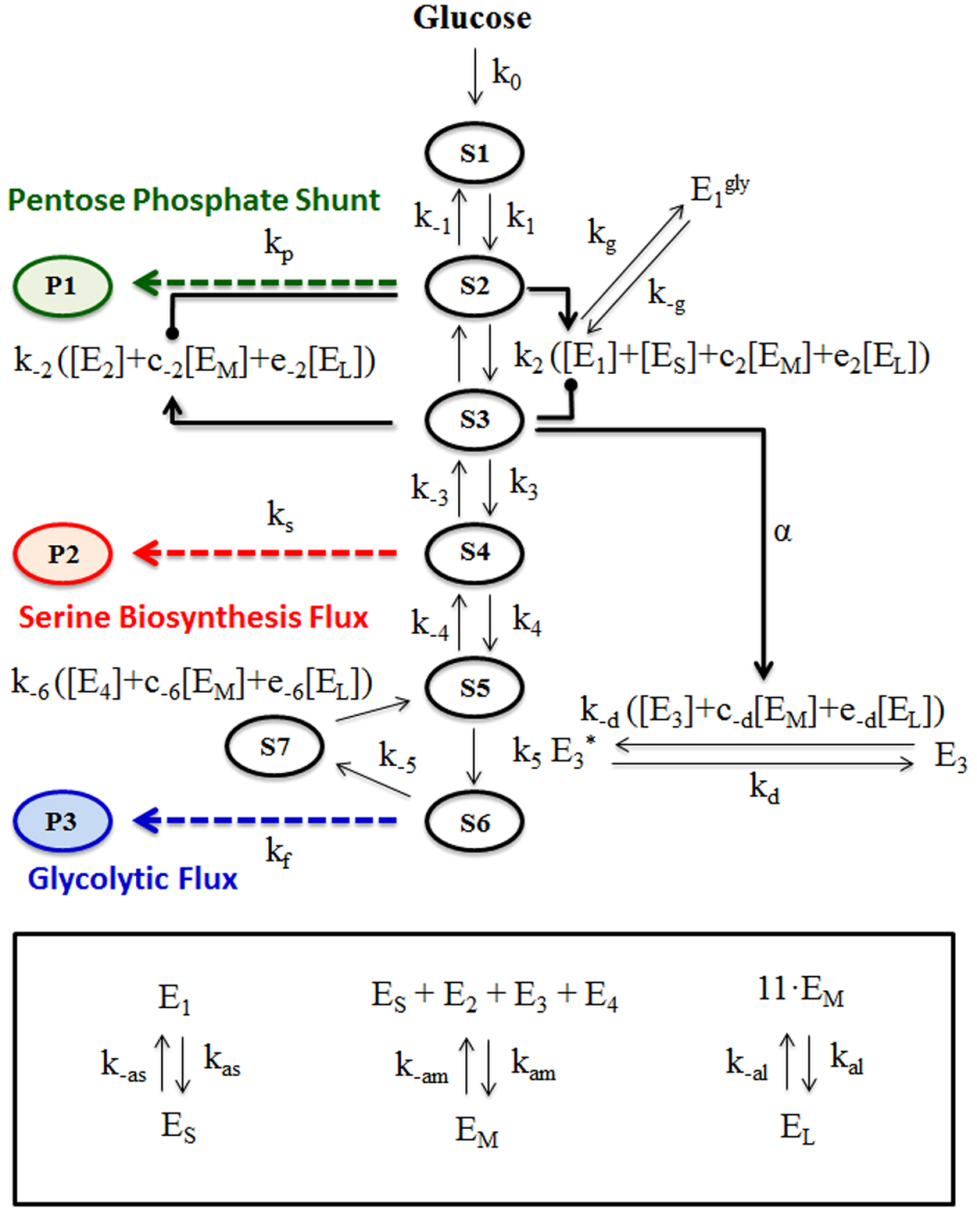
Simplified glucose metabolism with multienzyme complexes. Seven metabolic intermediates are involved in the pathway: *S*_1_ represents glucose, *S*_2_ is fructose-6-phosphate, *S*_3_ is fructose-1,6-bisphosphate, *S*_4_ is 3-phosphoglycerate, *S*_5_ is phosphoenolpyruvate, *S*_6_ is pyruvate, and *S*_7_ is oxaloacetate. Four rate-limiting enzymes are *E*_1_ (phosphofructokinase 1, PFK), *E*_2_ (fructose-1,6-bisphosphatase, FBPase), *E*_3_ (pyruvate kinase M2 dimer, PKM2), and *E*_4_ (phosphoenolpyruvate carboxykinase 1, PEPCK1). Pyruvate kinase M2 catalyzes conversion from *S*_5_ to *S*_6_ when it becomes a tetramer (${E}_{3}^{\ast }$). On the other hand, phosphofructokinase 1 is inactivated after post-translational glycosylation (*E*_1_^*gly*^). PFK forms three differently sized clusters: *E*_*S*_, *E*_*M*_, and *E*_*L*_ represent small-, medium- and large-sized clusters, where *E*_*M*_ and *E*_*L*_ are multienzyme complexes. To measure the direction of glucose flux, we denote three metabolic products as *P*_1_, *P*_2_, and *P*_3_, which represent metabolic outcomes of the pentose phosphate pathway, serine biosynthesis and the downstream of glycolysis. All the used parameters are summarized in Tables 1 and 2.

We develop a model to investigate how the size of glucosome clusters affects the direction of glucose flux in cancer environment as an indication of the metabolic activity of glucosome clusters in various sizes. Our model involves 7 metabolic intermediates (*S*_*i*_), 9 enzyme-associated species (*E*_*i*_) with various forms by which enzyme activities of the rate limiting steps are regulated, 3 metabolic products (*P*_*i*_), and 28 reactions (Fig. 1 and Tables 1 and 2). Among the fourteen enzymes participating in glycolysis and/or gluconeogenesis, we only focus on the enzyme activities of the glucosome members (i.e., PFKL, FBPase, PKM2, and PEPCK1), which are hypothesized to have different activity levels in a cluster size-dependent manner. In Fig. 1, we present our simplified metabolic pathway with the enzymes involved in the glucosome. All chemical species and reaction rate constants in our pathway are introduced in Tables 1 and 2. An ordinary differential equations (ODE) model was used to describe temporal dynamics of the metabolic network involving enzymatic reactions associated with glucose metabolism. Temporal concentration changes in each metabolic intermediate are determined by propensity functions of the reactions where the intermediate is produced or consumed. Table 3 summarizes the propensity function *R*_*i*_ for the *i*th reaction. In Table 3, most of the reactions are modeled by the law of mass action, in which the reaction rate is proportional to the substrate concentrations. The allosteric regulations of metabolic enzymes by metabolites are modeled as Michaelis-Menten kinetics, and their propensity functions include rational functions of the substrate concentrations to describe allosteric effects of the corresponding enzymes. Table 4 summarizes a set of all ODEs used in our model.

**Table 1:** The initial conditions used in the mathematical model for glucose metabolic pathway.

Variables	Chemical species	Values (non-dimensional)
*S*_1_(0)	Glucose	0.01
*S*_2_(0)	Fructose-6-Phosphate	0.01
*S*_3_(0)	Fructose-1,6-Bisphosphate	0.01
*S*_4_(0)	3-Phosphoglycerate	0.01
*S*_5_(0)	Phosphoenolpyruvate	0.01
*S*_6_(0)	Pyruvate	0.01
*S*_7_(0)	Oxaloacetate	0.01
*E*_1_(0)	Phosphofructokinase 1	99.99
*E*_2_(0)	Fructose-1,6-Bisphosphatase	100
*E*_3_(0)	Pyruvate Kinase M2 dimers	99.99
*E*_4_(0)	Phosphoenolpyruvate Carboxykinase 1	100
*E*_*S*_(0)	Small-sized enzyme clusters	0
*E*_*M*_(0)	Medium-sized enzyme clusters	0
*E*_*L*_(0)	Large-sized enzyme clusters	0
${E}_{3}^{\ast }(0)$	Pyruvate Kinase M2 tetramers	0.01
${E}_{1}^{gly}(0)$	Glycosylated Phosphofructokinase	0.01
*P*_1_(0)	Pentose Phosphate Shunt	0.01
*P*_2_(0)	Serine Biosynthesis Flux	0.01
*P*_3_(0)	Glycolytic Flux	0.01

**Table 2:** The rate constants used in the mathematical model for glucose metabolic pathway.

Parameters	Rates	Values (non-dimensional)
*k*_0_	Glucose production	10
*k*_1_, *k*_−1_	Conversion to (from) Fructose-6-Phosphate	10, 10
*k*_2_, *k*_−2_	Conversion to (from) Fructose-1,6-Bisphosphate	40, 7
*k*_3_, *k*_−3_	Conversion to (from) 3-Phophoglycerate	10, 10
*k*_4_, *k*_−4_	Conversion to (from) Phosphoenolpyruvate	14, 7
*k*_5_	Conversion to Pyruvate	1
*k*_−5_	Conversion to Oxaloacetate	10
*k*_−6_	Conversion to Phosphoenolpyruvate from Oxaloacetate	10
*k*_*as*_, *k*_−*as*_	Small enzyme cluster association/disassociation	10, 10
*k*_*am*_, *k*_−*am*_	Medium enzyme cluster association/disassociation	10, 10
*k*_*al*_, *k*_−*al*_	Large enzyme cluster association/disassociation	10, 10
*k*_*g*_, *k*_−*g*_	Phosphofructokinase glycosylation (de-glycosylation)	1, 1
*k*_*d*_, *k*_−*d*_	Conversion of Pyruvate Kinase M2 from (to) tetramer to (from) dimer	1, 1
*k*_*p*_	Pentose Phosphate Shunt	5
*k*_*s*_	Serine Biosynthesis Shunt	5
*k*_*f*_	Glycolytic Flux	5
*δ*_*p*_	Degradation of the Pentose Phosphate Flux	0.5
*δ*_*s*_	Degradation of Serine Biosynthesis Flux	0.5
*δ*_*f*_	Degradation of Glycolytic Flux	0.5
*c*_2_	Activation of conversion to Fructose-1,6-Bisphosphate by medium enzyme clusters	0.2
*c*_−2_	Activation of conversion from Fructose-1,6-Bisphosphate by medium enzyme clusters	10
*c*_−6_	Activation of conversion to Phosphoenolpyruvate from Oxaloacetate by medium enzyme clusters	10
*c*_−*d*_	Activation of conversion from Pyruvate Kinase M2 dimers to Pyruvate Kinase M2 tetramers by medium enzyme clusters	0.1
*e*_2_	Activation of conversion to Fructose-1,6-Bisphosphate by large enzyme clusters	2.5
*e*_−2_	Activation of conversion from Fructose-1,6-Bisphosphate by large enzyme clusters	0.1
*e*_−6_	Activation of conversion to Phosphoenolpyruvate from Oxaloacetate by large enzyme clusters	10
*e*_−*d*_	Activation of conversion from Pyruvate Kinase M2 dimers to Pyruvate Kinase M2 tetramers by large enzyme clusters	0.05
*α*	Acceleration of Fructose-1,6-Bisphosphate on Pyruvate Kinase M2 association	1
*K*_1_	Allosteric inhibition by Fructose-1,6-Bisphosphate	1
*K*_2_	Allosteric inhibition by Fructose-6-Phosphate	1
*K*_3_	Allosteric activation by Fructose-1,6-Bisphosphate	1

**Table 3:** Propensities of 28 reactions in the glucose metabolic pathway.

Reaction	Propensity	Reaction	Propensity	Reaction	Propensity
*R*_1_	*k*_0_	*R*_11_	*k*_−5_ *S*_6_	*R*_21_	${k}_{d}{E}_{3}^{\ast }$
*R*_2_	*k*_1_ *S*_1_	*R*_12_	*k*_−6_(*E*_4_ + *c*_−6_*E*_*M*_ + *e*_−6_*E*_*L*_)*S*_7_	*R*_22_	*k*_ −_ _*d*_ (*E*_3_ + *c* _−__*d*_*E*_*M*_ + *e*_ −__*d*_ *E*_*L*_) (1 + *α*(*S*_3_)/(*S*_3_ + *K*_3_))
*R*_3_	*k*_−1_ *S*_2_	*R*_13_	*k*_*as*_ *E*_1_	*R*_23_	*k*_p_ *S*_2_
*R*_4_	*k*_2_(*E*_1_ + *E*_*S*_ + *c*_2_*E*_*M*_ + *e*_2_*E*_*L*_)*S*_2_(*K*_1_)/(*K*_1_ + *S*_3_)	*R*_14_	*k*_−*as*_ *E*_*S*_	*R*_24_	*k*_s_ *S*_4_
*R*_5_	*k*_−2_(*E*_2_ + *c*_−2_*E*_*M*_ + *e*_−2_*E*_*L*_)*S*_3_(*K*_2_)/(*K*_2_ + *S*_2_)	*R*_15_	*k*_*am*_ *E*_*S*_ *E*_2_ *E*_3_ *E*_4_	*R*_25_	*k*_f_ *S*_6_
*R*_6_	*k*_3_ *S*_3_	*R*_16_	*k*_−*am*_ *E*_*M*_	*R*_26_	*δ*_p_ *P*_1_
*R*_7_	*k*_−3_ *S*_4_	*R*_17_	*k*_a__l_*(E*_*M*_)^11^	*R*_27_	*δ*_s_ *P*_2_
*R*_8_	*k*_4_ *S*_4_	*R*_18_	*k*_−__al_ *E*_*L*_	*R*_28_	*δ*_f_ *P*_3_
*R*_9_	*k*_−4_ *S*_5_	*R*_19_	*k*_g_ *E*_1_		
*R*_10_	${k}_{5}{E}_{3}^{\ast }{S}_{5}$	*R*_20_	${k}_{-g}{E}_{1}^{gly}$

**Table 4:** A system of ODEs for metabolic intermediates, enzymes and their clusters, and metabolic products.

Equations
*S* = (*S*_1_, *S*_2_, *S*_3_, *S*_4_, *S*_5_, *S*_6_, *S*_7_)^*T*^, $$E={({E}_{1},{E}_{2},{E}_{3},{E}_{4},{E}_{S},{E}_{M},{E}_{L},{E}_{3}^{\ast },{E}_{1}^{gly})}^{T}$$, *P* = (*P*_1_, *P*_2_, *P*_3_)^*T*^
$$\frac{dS}{dt}={\nu }_{S}{ {\mathcal R} }_{S}(S,E)$$, $$\frac{dE}{dt}={\nu }_{E}{ {\mathcal R} }_{E}(S,E)$$, $$\frac{dP}{dt}={\nu }_{P}{ {\mathcal R} }_{P}(S,P)$$
$${ {\mathcal R} }_{S}(S,E)={[{R}_{1},{R}_{2},{R}_{3},{R}_{4},{R}_{5},{R}_{6},{R}_{7},{R}_{8},{R}_{9},{R}_{10},{R}_{11},{R}_{12},{R}_{23},{R}_{24},{R}_{25}]}^{{\rm{T}}}$$ $${ {\mathcal R} }_{E}(S,E)={[{R}_{13},{R}_{14},{R}_{15},{R}_{16},{R}_{17},{R}_{18},{R}_{19},{R}_{20},{R}_{21},{R}_{22}]}^{{\rm{T}}}$$ $${ {\mathcal R} }_{P}(S,P)={[{R}_{23},{R}_{24},{R}_{25},{R}_{26},{R}_{27},{R}_{28}]}^{{\rm{T}}}$$
$${\nu }_{S}=[\begin{array}{ccccccccccccccc}1 & -1 & 1 & 0 & 0 & 0 & 0 & 0 & 0 & 0 & 0 & 0 & 0 & 0 & 0\\ 0 & 1 & -1 & -1 & 1 & 0 & 0 & 0 & 0 & 0 & 0 & 0 & -1 & 0 & 0\\ 0 & 0 & 0 & 1 & -1 & -1 & 1 & 0 & 0 & 0 & 0 & 0 & 0 & 0 & 0\\ 0 & 0 & 0 & 0 & 0 & 1 & -1 & -1 & 1 & 0 & 0 & 0 & 0 & -1 & 0\\ 0 & 0 & 0 & 0 & 0 & 0 & 0 & 1 & -1 & -1 & 0 & 1 & 0 & 0 & 0\\ 0 & 0 & 0 & 0 & 0 & 0 & 0 & 0 & 0 & 1 & -1 & 0 & 0 & 0 & -1\\ 0 & 0 & 0 & 0 & 0 & 0 & 0 & 0 & 0 & 0 & 1 & -1 & 0 & 0 & 0\end{array}]$$ $${\nu }_{E}=[\begin{array}{cccccccccc}-1 & 1 & 0 & 0 & 0 & 0 & -1 & 1 & 0 & 0\\ 0 & 0 & -1 & 1 & 0 & 0 & 0 & 0 & 0 & 0\\ 0 & 0 & -1 & 1 & 0 & 0 & 0 & 0 & 1 & -1\\ 0 & 0 & -1 & 1 & 0 & 0 & 0 & 0 & 0 & 0\\ 1 & -1 & -1 & 1 & 0 & 0 & 0 & 0 & 0 & 0\\ 0 & 0 & 1 & -1 & -11 & 11 & 0 & 0 & 0 & 0\\ 0 & 0 & 0 & 0 & 1 & -1 & 0 & 0 & 0 & 0\\ 0 & 0 & 0 & 0 & 0 & 0 & 0 & 0 & -1 & 1\\ 0 & 0 & 0 & 0 & 0 & 0 & 1 & -1 & 0 & 0\end{array}]$$ $${\nu }_{P}=[\begin{array}{cccccc}1 & 0 & 0 & -1 & 0 & 0\\ 0 & 1 & 0 & 0 & -1 & 0\\ 0 & 0 & 1 & 0 & 0 & -1\end{array}]$$

We also provide reaction rate constants and initial values for the intermediate concentrations used in our model (Tables 1 and 2). We determined the parameters in Tables 1 and 2 using the numerical simulation of the model to be consistent with the qualitative changes in glucose flux in the literature^15^. As shown in Table 1, we assumed that initial concentrations of metabolic intermediates (*S*_*i*_), products (*P*_*i*_), PKM2 tetramers (E_3_*) and glycosylated PFKL (E_1_^gly^) are at the basal levels and set them as 0.01. We also assumed that no multienzyme complex (E_*S/M/L*_) is formed at the beginning of simulation. An enzyme concentration of each glucosome member is assumed to be conserved, setting as 100. Assuming that all reaction rate constants for the enzymes are in the same order of magnitude, the reaction rate constants of metabolic enzymes are set to 10 as shown in Table 2. However, we slightly modified the values for *k*_2_, *k*_−2_, *k*_4_, and *k*_−4_ so that glycolysis becomes dominant relative to the metabolic shunt to the pentose phosphate pathway and/or serine biosynthesis in the absence of enzyme clusters (i.e., *P*_3_ ≫ *P*_1_, *P*_2_). Lastly, we assumed that all the metabolic products are produced (*k*_*p/s/f*_) and degraded (*δ*_*p/s/f*_) in the same rates, thus setting them to 5 and 0.5, respectively. Note that we provided both forward and backward reaction rate constants in Table 2, which are separated by comma.

Next, different levels of the enzyme activity depending on the size of glucosome clusters are expressed in terms of the parameters *c*_*i*_ (for medium-sized enzyme clusters) and *e*_*i*_ (for large-sized enzyme clusters) in Table 2. Based on our hypothesis that the medium-sized enzyme clusters promote glucose shunt to the pentose phosphate pathway^4^, we decelerated glycolysis (*c*_2_, *c*_−*d*_ ≪ 1) and accelerated gluconeogenesis (*c*_−2_, *c*_−6_ ≫ 1). Similarly, based on our hypothesis that the large-sized enzyme clusters will shunt glucose flux to serine biosynthesis^4^, we changed parameters to direct glucose flux toward serine production (*e*_2_, *e*_−6_ ≫ 1 , and *e*_−2_, *e*_−*d*_ ≪ 1). An enzyme activity in the small-sized clusters is assumed similar to the activity of a freestanding enzyme because the small-sized clusters formed by PFKL do not represent a multienzyme complex^4^. Using the initial values and the reaction rate constants shown in Tables 1 and 2, our mathematical model was simulated using MATLAB to compute temporal changes of glucose flux in different scenarios at the subcellular level. Note that our MATLAB simulation codes are provided in the supplementary document.

Importantly, we have also performed sensitivity analysis of the parameters to investigate how the concentrations of three metabolic products (P_*i*_) are affected by small changes in each parameter that we have incorporated in our model. In the sensitivity analysis, we have used the method of partial rank correlation coefficient (PRCC)^16^ to evaluate the level of the linear association between the input values of the parameters and the output concentrations of the metabolic products. To perform the sensitivity analysis, we modified the publically available MATLAB codes which are developed by Kirschner and coworker^16^. The default parameter values were set as the values used in our model, and we defined an interval for each parameter from half of the default value to twice of the default value. Then, we used Latin hypercube sampling^16^ to choose each parameter value in the corresponding interval by assuming that each parameter is uniformly distributed in the interval. Afterward, the sampled parameter values were used to calculate the concentrations of the metabolic products at the end time of the simulation. We repeated the sampling process described above 20,000 times and calculated the Spearman correlation coefficients, which correspond to PRCCs.

### Experimental Methods

#### Materials

The plasmid expressing human PFKL with a monomeric form of enhanced green fluorescent protein (mEGFP) was prepared previously (PFKL-mEGFP)^4^. We have employed PFKL-mEGFP as a marker of the glucosome. 2-Deoxyglucose was purchased from Sigma.

#### Cell Culture

Human breast carcinoma cells, Hs578T (HTB-126), were obtained from the American Type Culture Collection (ATCC). Hs578T cells were maintained in the Roswell Park Memorial Institute 1640 medium (RPMI1640, Mediatech, Cat# 10-040-CV) supplemented with 10% dialyzed fetal bovine serum (dFBS, Atlanta Biological, Cat# S12850) and 50 µg/mL gentamycin sulfate.

#### Transfection

To prepare cells for transfection and subsequent imaging, Hs578T cells were gently removed from the culture flask by replacing the culture medium with Trypsin-EDTA solution (Corning, Cat# 25-053-Cl). Fresh, antibiotic-free growth media was subsequently used to harvest and resuspend the cells, followed by plating on glass-bottomed 35 mm Petri dishes (MatTek). When the confluency became ~70–90% on the following day, the cells were transfected with Lipofectamine 2000 (Invitrogen) using the Opti-MEM-I reduced serum media (Opti-MEM-I; Gibco, Cat# 11058). The Opti-MEM-I medium was then exchanged with the fresh antibiotic-free growth medium after a 5 h incubation (37 °C, 5% CO_2_, and 95% humidity), followed by ~18–24 h incubation in the CO_2_ incubator at 37 °C.

#### Fluorescence Live-cell Imaging

On the day of imaging (~18–24 h post-transfection), cells were washed with imaging solution (20 mM HEPES (pH 7.4), 135 mM NaCl, 5 mM KCl, 1 mM MgCl_2_, 1.8 mM CaCl_2_, and 5.6 mM glucose) for three 10 min incubations, followed by a ~1–2 h incubation at ambient temperature. All samples were then imaged at ambient temperature (~25 °C) with a 60× 1.45 NA objective (Nikon CFI Plan Apo TIRF) using a Photometrics CoolSnap EZ monochrome CCD camera on a Nikon Eclipse Ti inverted C2 confocal microscope. Epifluorescence imaging was carried out using the following filter set from Chroma Technology; mEGFP detection by a set of Z488/10-HC cleanup, HC TIRF Dichroic and 525/50-HC emission filter.

For cell-based high-content imaging assays, 2-deoxyglucose (25 mM) was added to Hs578T cells after washing three times with the imaging solution. Images showing PFKL-mEGFP clusters were acquired before and after cells were incubated with 2-deoxyglucose at various time points (up to 6 hours). Control experiments were also carried out with 167 μL of vehicle (i.e., water). To ensure reproducibility, our experiments were repeated at least five times over the course of a few months. Statistical analysis was performed using two-sample two-tail *t*-tests.

#### Cluster Size Analysis

Cluster size analysis was accomplished using the *ImageJ* processing software (National Institutes of Health) as we have done before^4^. Briefly, fluorescent wide-field images were processed through *ImageJ* using a custom script and macro that automates the counting of fluorescent clusters using its built-in module, so-called robust automatic threshold selection (RATS). In this analysis, the images were scaled according to the pixel size of the microscope (i.e., 0.12 µm/pixel) before the default parameters for RATS (i.e., noise threshold = 25, λ factor = 3) were used in this analysis. Once fluorescent clusters were selected from an image, the particle analysis module was applied to attain both the number and area of fluorescent clusters within an image. This process was repeated for all subsequent cell images. The operator then evaluated the original cell images against the particle mask to eliminate data in which more than one cluster was counted as a single particle.

## Results

### Modeling Glucose Metabolism with Multienzyme Complexes

Glucose metabolism consists of glycolysis and gluconeogenesis where three irreversible reactions are involved in glycolysis, four irreversible reactions in gluconeogenesis, and seven reversible reactions in both pathways. In our model, we simplify the 14-step pathway by condensing a few reversible steps into one conversion (Fig. 1). Accordingly, we omit some of the metabolic intermediates such as glucose-6-phosphate, dihydroxyacetone phosphate, glyceraldehyde-3-phosphate, 1,3-bisphosphoglycerate, and 2-phosphoglycerate because they are not directly shuttled to other metabolic pathways. In addition, three metabolic products (*P*_*i*_) represent three metabolic fates of glycolytic metabolites. Importantly, we focus on four cytoplasmic enzymes which spatially organize into multienzyme clusters (i.e., glucosome clusters)^4^: PFKL, FBPase, PKM2, and PEPCK1. Note that the other ten enzymes are not cytoplasmic or rate-determining in glucose metabolism.

In addition, we incorporated cancer-relevant mechanisms of the four enzymes forming the glucosome. First, pyruvate kinase catalyzes conversion from phosphoenolpyruvate to pyruvate. The M2 isoform of pyruvate kinase (PKM2) is predominantly expressed in cancer cells while suppressing the expression of pyruvate kinase M1 isoform (PKM1)^17^. PKM2 plays an important role in aerobic glycolysis as a dimer by impairing pyruvate production, thus redirecting glucose flux into serine biosynthesis^18,19^. However, a tetrameric form of PKM2 possesses its canonical glycolytic activity in production of pyruvate in cancer cells. We take into account the dimer-tetramer conversion as part of the regulation of glucose metabolism (Fig. 1). Second, PFK catalyzes the conversion of fructose-6-phosphate to fructose-1,6-bisphosphate. Particularly, liver-type PFK (PFKL) is glycosylated in several types of cancer cells under hypoxia conditions, diverting the direction of glucose flux into the pentose phosphate pathway^20^. Our model includes the impact of glycosylation on PFKL as part of the regulation of its metabolic activity in glycolysis. Third, cytoplasmic PEPCK1 plays an essential role in the first step of gluconeogenesis^21^. Recently, a new role of PEPCK1 in tumor cell proliferation was discovered in certain types of cancer cells by controlling carbon metabolic flux through the TCA cycle^22^. However, a molecular level mechanism of PEPCK1 in cancer cells (e.g., post-translational modification-dependent activity or oligomerization-dependent activity) is not firmly established yet to be included in our model. Fourth, FBPase catalyzes the conversion of fructose-1,6-bisphosphate to fructose-6-phosphate. Since FBPase catalyzes the backward reaction of PFK, the activities of PFK and FBPase are reciprocally regulated by various allosteric metabolites. Similarly, an antagonistic role of FBPase for glycolytic flux was validated to play a role in kidney cancer progression^23^. However, other than the well-established allosteric regulation, cancer-specific alteration of FBPase is not established yet to be included in our model.

Unlike many previous mathematical models for glycolysis, we take into account the recent discovery of the glucosome being formed in various sizes in cancer cells. Briefly, various sizes of PFKL clusters in cancer cells are categorized into three subclasses; small, medium and large-sized cluster^4^. Small-sized clusters formed by PFKL are single-enzyme assemblies whereas medium- and large-sized clusters represent spatial organizations of multienzyme complexes. When cancer cells display small-sized clusters, the given cells barely include the other sizes of clusters. However, cancer cells showing medium- and large-sized clusters contain smaller sized clusters. Our cluster size analysis has also revealed that HeLa cells with medium-sized clusters show ~86.1 clusters per cell whereas HeLa cells showing large-sized clusters exhibit ~5.6 large-sized clusters and ~25.1 medium-sized clusters per cell^4^. Accordingly, we assume that 11 medium-sized clusters might form one large cluster in the cytoplasm of cancer cells in our model.

### Glucose Flux Analysis at Subcellular Levels

**Figure 2:**
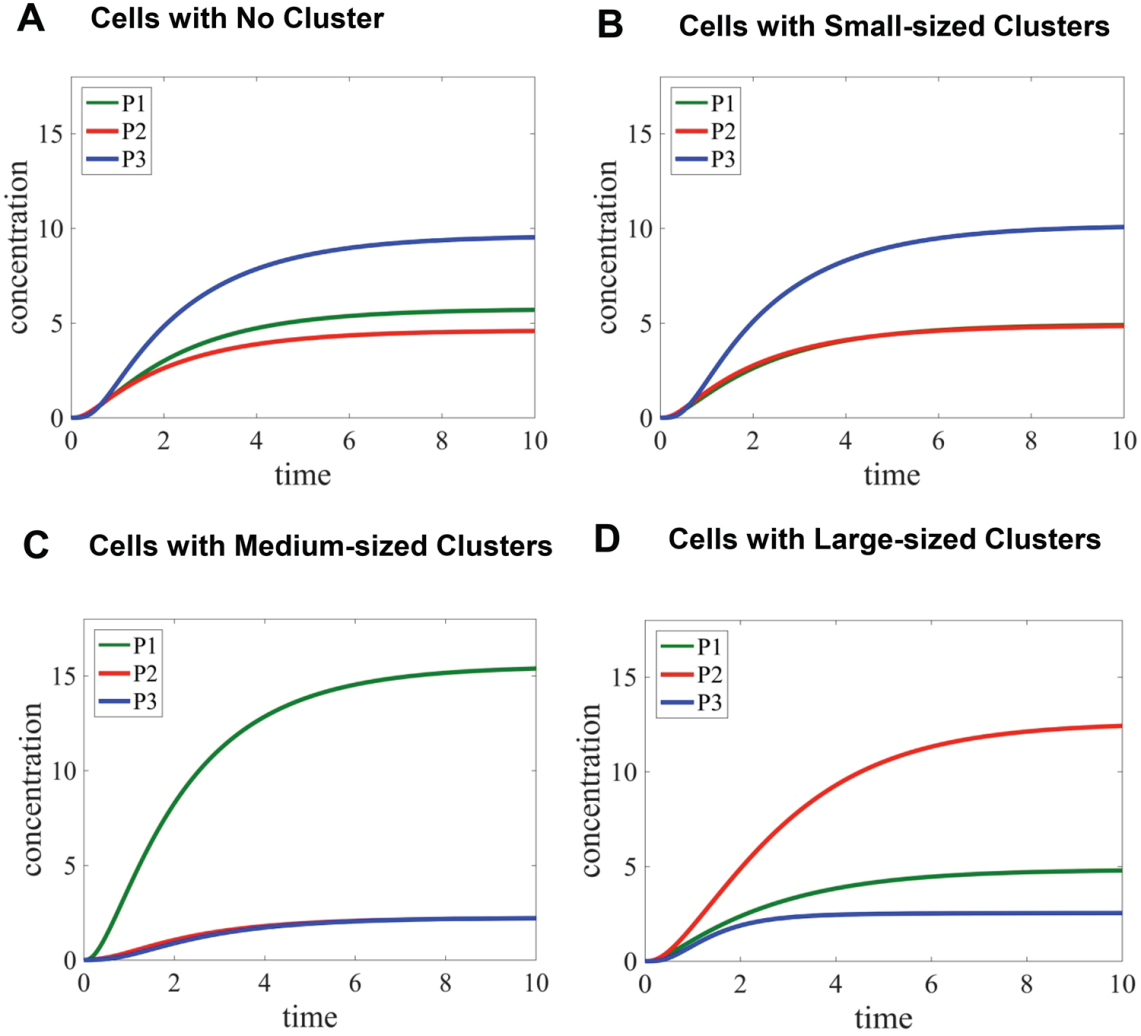
Glucose flux analysis at subcellular levels. Time-dependent changes of metabolic product concentrations (*P*_1_*, P*_2_ and *P*_3_) in single cells are graphed for: (**A**) cells with no spatial organization, (**B**) cells with small-sized PFKL clusters which indicate single enzyme assemblies, (**C**) cells with medium-sized multienzyme complexes, and (**D**) cells with large-sized multienzyme complexes in the presence of smaller clusters. Arbitrary units (a.u.) are used to show relative concentrations or time during our model simulation. *P*_1_, *P*_2_, and *P*_3_ represent metabolic outcomes of the pentose phosphate pathway, serine biosynthesis and the downstream of glycolysis, respectively.

In Fig. 2, metabolic product concentrations are shown in different experimental conditions, which are computed by numerically solving the mathematical model given in Tables 3 and 4. The model results in time-dependent changes of three metabolic products: *P*_1_ represent a metabolic product of the pentose phosphate shunt (green line), *P*_2_ is a metabolic product of serine biosynthesis (red line), and *P*_3_ indicates a metabolic product of the downstream of glycolysis (blue line). In addition, the activity of non-clustering PFKL or clustered PFKL into small sizes are anticipated to be similar in our model. For the simulation of the cases with no cluster or only small-sized PFKL clusters, we set all the parameters related to the formation of medium or large-sized enzyme complexes as zero (i.e., *k*_*am*_, *k*_−*am*_, *k*_*al*_, *k*_−*al*_, *c*_*i*_, and *e*_*i*_). Please, note that the parameters for small-sized PFKL clusters (i.e., *k*_*as*_ and *k*_−*as*_) were also set to zero when we simulated  the case showing no cluster. Consequently, our simulation for no cluster or only small-sized PFKL clusters showed the high level of *P*_3_ relative to those of *P*_1_ and *P*_2_ (Fig. 2A and B), indicating that most of glucose flux flows to glycolysis to produce pyruvate and beyond. However, when the rate-limiting enzymes in glucose metabolism are spatially organized into medium-sized glucosome clusters, the enzymes in medium-sized clusters may have different levels of catalytic activity. Because the increased flux of the pentose phosphate shunt is correlated with the high level of medium-sized clusters in cancer cells^4^, our assumption of *c*_2_, *c*_−*d*_ ≪ 1 and *c*_−2_, *c*_−6_ ≫ 1 resulted in a significant increase of *P*_1_ but decreased the concentrations of *P*_2_ and *P*_3_ (Fig. 2C). In this case, we set the values of *k*_*al*_, *k*_−*al*_, and *e*_*i*_ as zero. Lastly, we considered all the rate-limiting enzymes being assembled into large-sized glucosome clusters in the presence of smaller clusters. Because the promotion of large-sized clusters in cancer cells is correlated with the increased flux of serine biosynthesis^4^, our assumption of *e*_2_, *e*_−6_ ≫ 1 and *e*_−2_, *e*_−*d*_ ≪ 1 along with the same assumption of *c*_*i*_ in the previous case resulted in the substantially increased level of *P*_2_ relative to the levels of *P*_1_ and *P*_3_ (Fig. 2D). Collectively, our mathematical model is developed to understand the cluster size-dependent changes of glucose flux in cancer cells.

### Sensitivity Analysis

**Figure 3:**
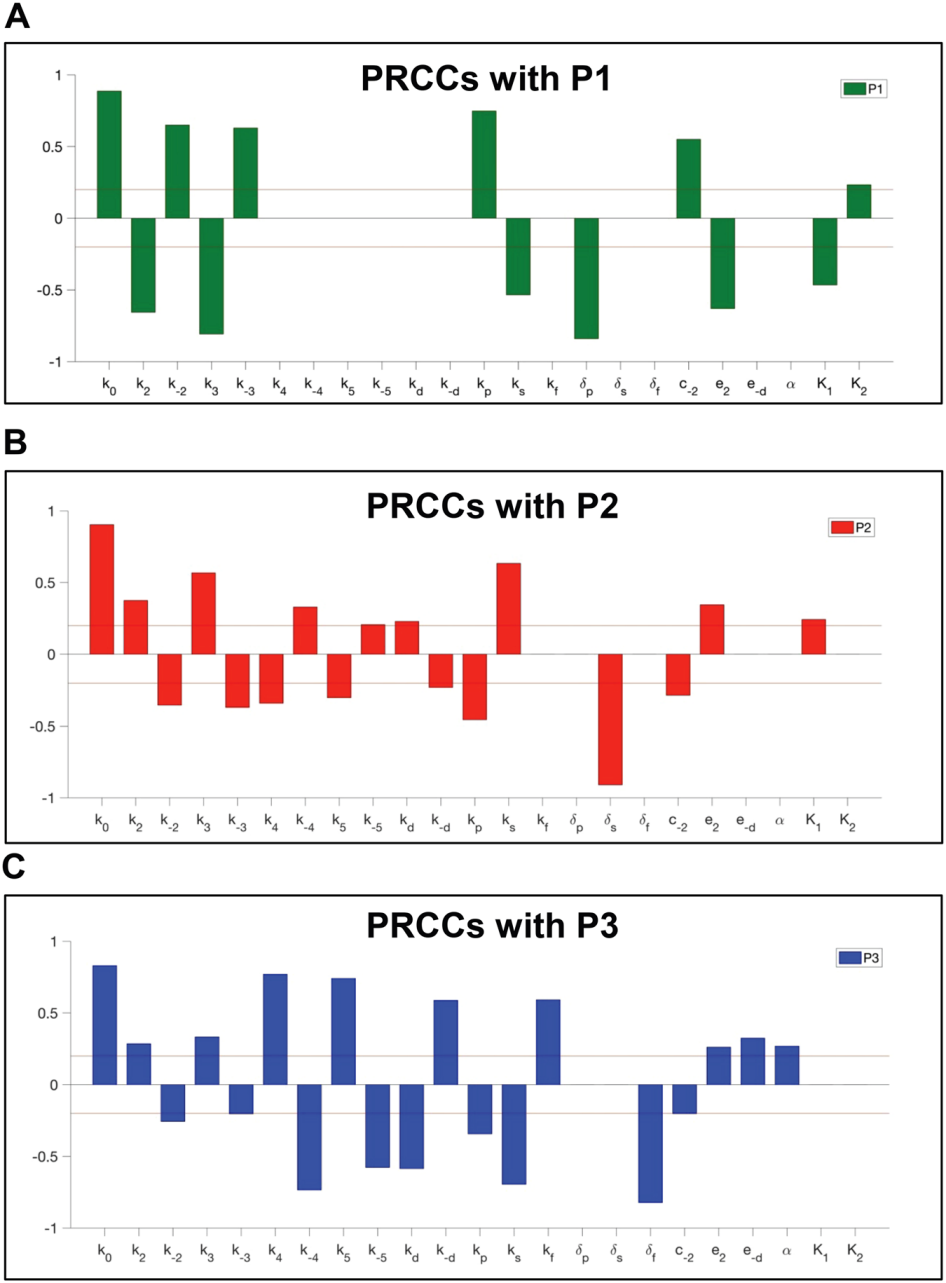
The partial rank correlation coefficients (PRCCs) between the ‘essential’ input parameters and the concentrations of metabolic products. The PRCCs at *time* = 10 are graphed to provide relative strengths of the correlations between the input parameters and the concentrations of metabolic products (*P*_1_, *P*_2_, and *P*_3_). The horizontal lines at ±0.2 indicate the thresholds we used to distinguish sensitive essential parameters from non-essential parameters. The PRCCs of all parameters are shown in supplementary figure S1. *P*_1_, *P*_2_, and *P*_3_ represent metabolic outcomes of the pentose phosphate pathway, serine biosynthesis and the downstream of glycolysis, respectively.

Next, we performed a sensitivity analysis by which we evaluated without bias how the input values of simulation parameters influence the production of specific metabolic flux (*P*_*i*_). According to the publically available MATLAB codes developed by Kirschner and coworker^16^, we modified to calculate PRCCs in our model (Figure S1). If the PRCC values are close to −1 or +1, the corresponding parameters have strong correlations with the formation of the given metabolic product. On the other hand, if the correlation coefficient is close to 0, there is no correlation between the input parameters and the given metabolic product. When the absolute values of the PRCCs are greater than ±0.2 and their *p*-values are less than 0.01, we defined the corresponding parameters as ‘sensitive’ parameters (Fig. 3) to determine which simulation parameters are critical for our model and how sensitive each parameter is to understand its contribution to the outcomes of glucose flux. Noticeably, the formation of each metabolic product was sensitive not only to the enzyme activities of the glucosome members but also the efficiencies of enzyme clustering into the certain sizes of glucosomes.

**Figure 4:**
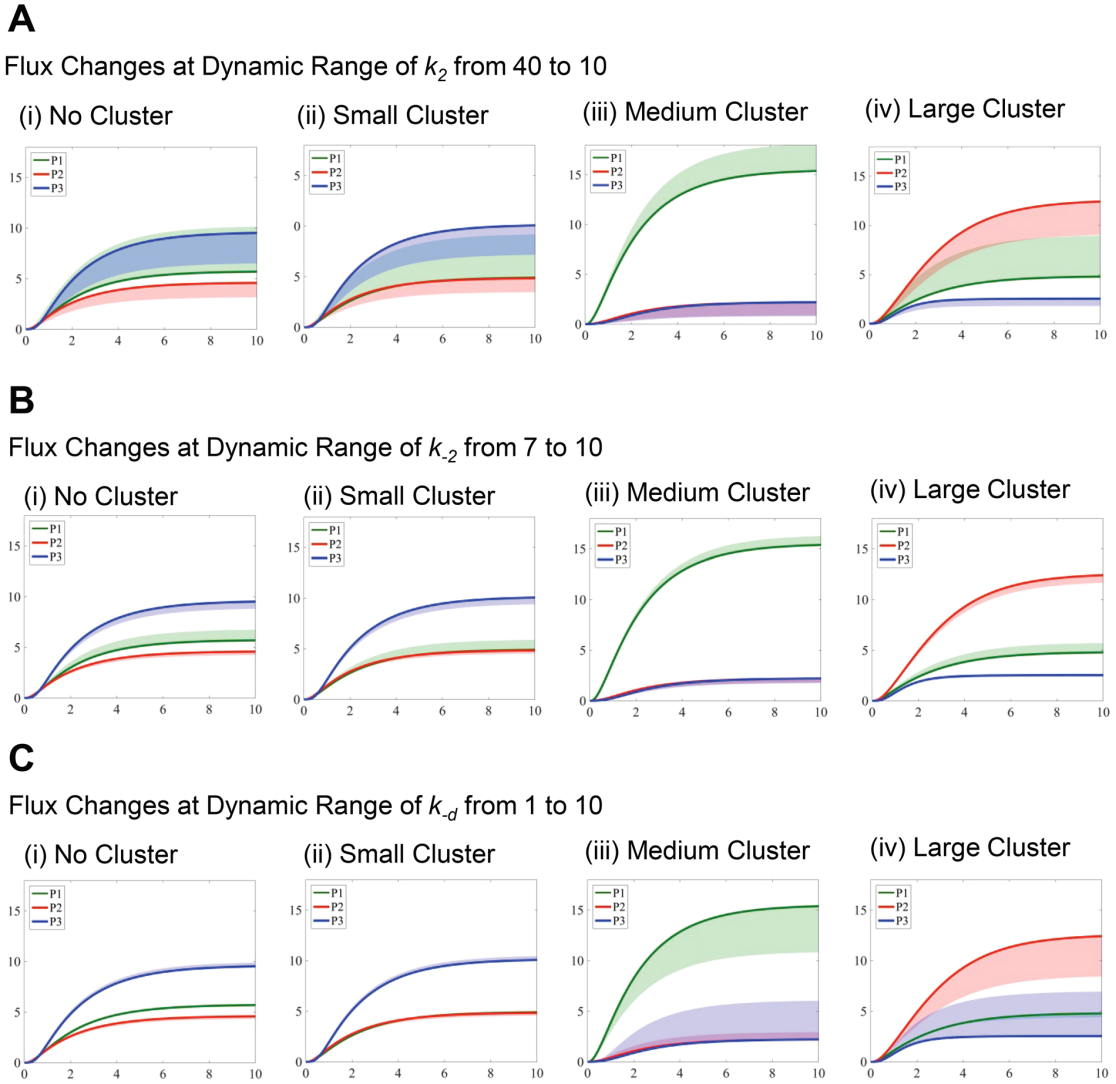
Simulated concentration changes of metabolic products with varying ranges of the catalytic activities of glucosome members. Time-dependent concentration changes of three metabolic products are expressed as shaded regions, when one ‘essential’ kinetic rate constant is perturbed for *k*_2_ ranging from 40 to 10 (**A**), *k*_−2_ ranging from 7 to 10 (**B**), and *k*_−*d*_ from 1 to 10 (**C**). In each case, time-dependent concentration changes of three metabolic products in single cells are graphed for (i) cells with no spatial organization, (ii) cells with small-sized PFKL clusters, (iii) cells with medium-sized multienzyme complexes, and (iv) cells with large-sized multienzyme complexes.

In Fig. 4, we further analyzed a few critical parameters that influence the enzyme activities of the glucosome members (i.e., *k*_2_, *k*_*−2*_, and *k*_−*d*_). Each selected parameter was perturbed in one direction while all other parameter values were fixed as default values as shown in Table 2. The changes in the metabolic product concentrations were then presented under four cases; (i) no spatial organization, (ii) small-sized PFKL clusters, (iii) medium-sized multienzyme complexes, and (iv) large-sized multienzyme complexes. Note that for the cases dealing with the medium-sized or large-sized complexes, the smaller-sized clusters are also formed in the model. When *k*_2_ was decreased from 40 to 10 (Fig. 4A), the conversion from *S*_2_ to *S*_3_ was reduced. We thus observed significant increase in *P*_1_ while decrease in *P*_2_ and *P*_3_. On the other hand, when *k*_−2_ was increased from 7 to 10 (Fig. 4B), a similar pattern was observed as in Fig. 4A although the changes were small due to the narrow ranges of the parameter. Finally, when *k*_−*d*_ was increased from 1 to 10 (Fig. 4C), glucose flux moved toward the production of *P*_3_, which resulted in significant increase in *P*_3_ and decrease in *P*_1_ or *P*_2_. Note that we did not observe much increase of *P*_3_ in the cases where no spatial organization or only small-sized PFKL clusters are dominant (Fig. 4C) because the flux was already committed toward the production of *P*_3_ in these cases. Collectively, the simulated flux changes reflect the changes of enzymatic activities involved in glucosome clusters.

**Figure 5:**
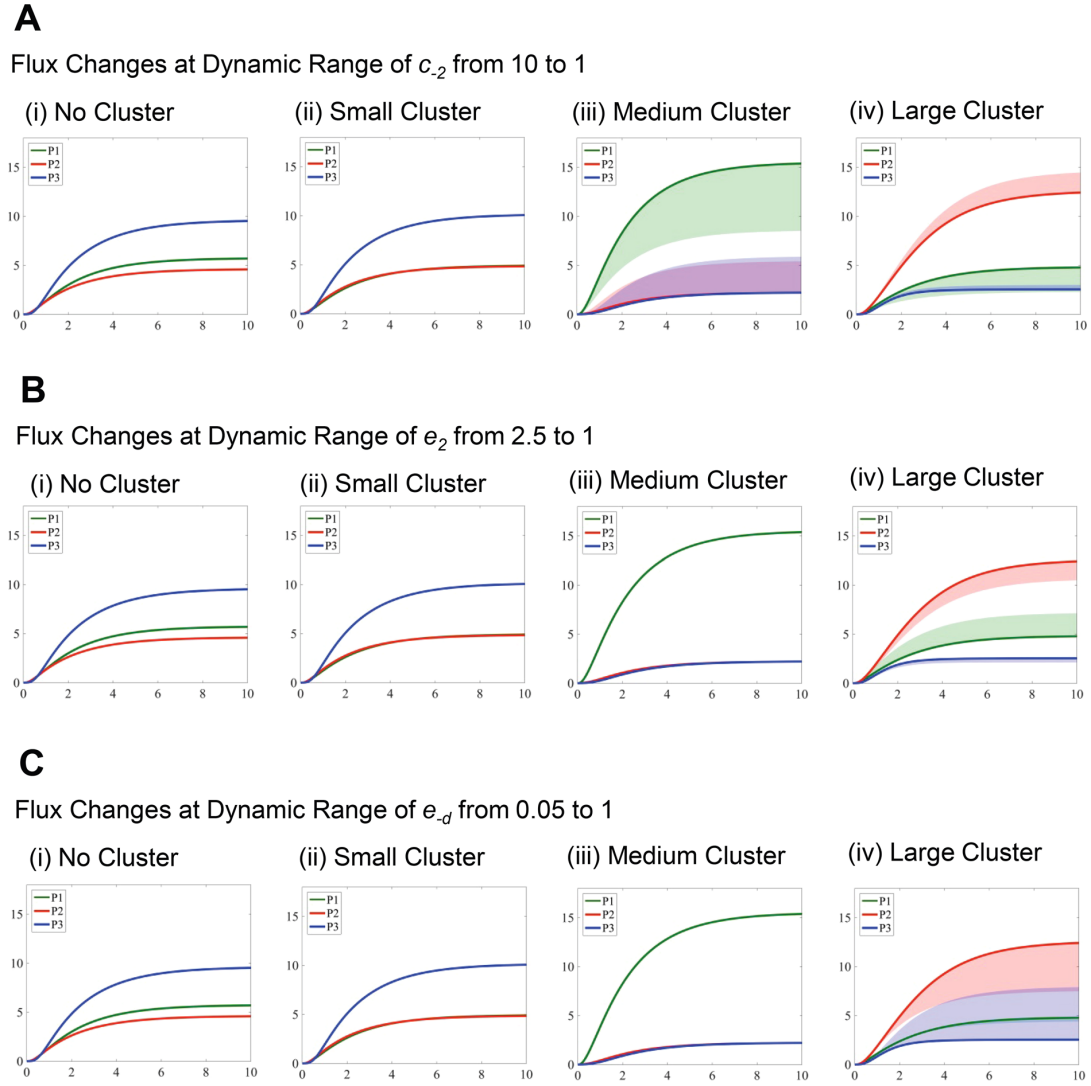
Simulated concentration changes of metabolic products with varying ranges of the clustering efficiency of glucosomes. Time-dependent concentration changes of three metabolic products are expressed as shaded regions, when one ‘essential’ clustering efficiency is perturbed for *c*_−2_ ranging from 10 to 1 (**A**), *e*_2_ from 2.5 to 1 (**B**), and *e*_−*d*_ from 0.05 to 1 (**C**). In each case, time-dependent concentration changes of three metabolic products in single cells are graphed for (i) cells with no spatial organization, (ii) cells with small-sized PFKL clusters, (iii) cells with medium-sized multienzyme complexes, and (iv) cells with large-sized multienzyme complexes.

In Fig. 5, we also analyzed more critical parameters that influence the efficiencies of enzyme clustering into the glucosomes (i.e., *c*_−2_, *e*_2_, and *e*_−*d*_). Similarly, each selected parameter was perturbed in one direction while fixing the other parameter values. The changes in the metabolic product concentrations were shown under the four cases as well. When *c*_−2_ was decreased from 10 to 1 (Fig. 5A), the level of *P*_1_ decreased and that of *P*_2_ increased significantly due to the decreased contribution of the medium-sized cluster to the conversion from *S*_3_ to *S*_2_. When *e*_2_ was decreased from 2.5 to 1 (Fig. 5B), the resulting effect was quite opposite from the one shown Fig. 5A because the decreased *e*_2_ reduces the contribution of the large-sized clusters to the conversion of *S*_2_ to *S*_3_. When *e*_−*d*_ was increased from 0.05 to 1 (Fig. 5C), the level of *P*_2_ decreased and that of *P*_3_ increased significantly with slight decrease in *P*_1_. This is due to the increased efficiency of the large-sized cluster on its contribution to the conversion of *E*_3_ to ${E}_{3}^{\ast }$.

### Metabolic Flux Analysis at Ensemble Levels

**Figure 6:**
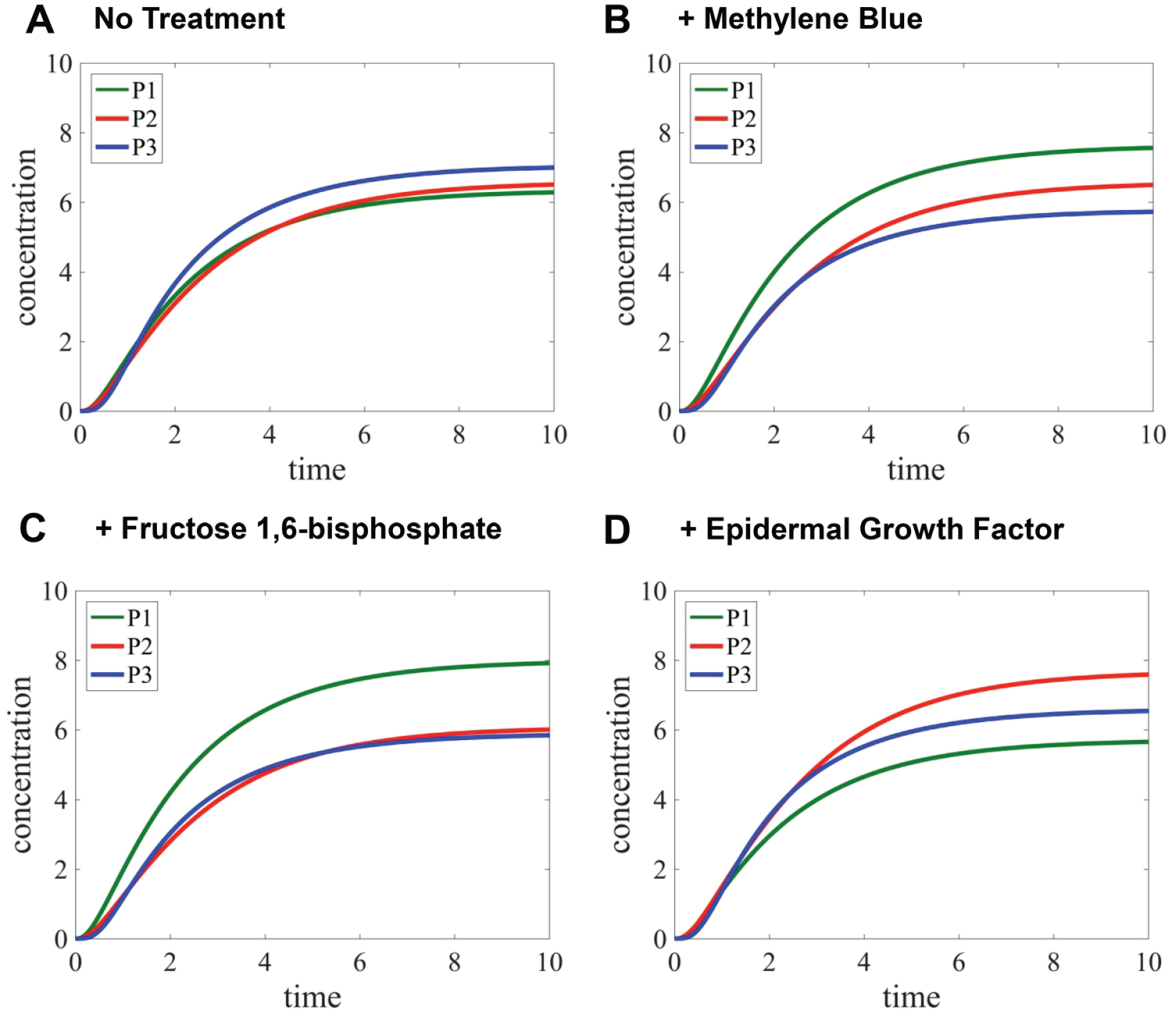
Metabolic flux analysis at ensemble levels. Time-dependent concentration changes of three metabolic products are simulated by our mathematical model in the four scenarios: cancer cells without a glucose flux regulator as a control (**A**), cancer cells that are treated with 5 nM methylene blue (**B**) or 15 mM fructose-1,6-bisphosphate (**C**), and cancer cells with 30 ng/ml epidermal growth factors (**D**). Note that human breast cancer cells (Hs578T) were cultured in the medium of RPMI1640 and 10% dialyzed FBS. *P*_1_, *P*_2_, and *P*_3_ represent metabolic outcomes of the pentose phosphate pathway, serine biosynthesis and the downstream of glycolysis, respectively.

We have further evaluated our model by quantifying the changes of glucose flux in the presence of methylene blue, fructose-1,6-bisphosphate or epidermal growth factor (EGF), which are known to regulate the direction of glucose flux at ensemble levels^24–29^. As shown in Table 5, we have counted the percentages of cells showing no cluster, small, medium, and large clusters in the absence and presence of the listed glucose flux regulators. In combination with our subcellular flux analysis (Fig. 2), we have computed the time-dependent changes of metabolic products (*P*_1_, *P*_2_, and *P*_3_) at an ensemble level in the four published cases^4^. To predict the levels of metabolic products at an ensemble level, we multiplied the levels of *P*_1_, *P*_2_, and *P*_3_ of the four cases shown in Fig. 2 by the corresponding proportions of the cells showing differently sized clusters. In a control population of Hs578T cells without any exogenous stimulus (Fig. 6A), the level of *P*_3_ appears to be slightly greater than those of *P*_1_ and *P*_2_. However, relative to Fig. 6A, the cancer cells that were treated with methylene blue (Fig. 6B) or fructose-1,6-bisphosphate (Fig. 6C) increase the level of *P*_1_ while decreasing the level of *P*_3_ at an ensemble level. On the other hand, Fig. 6D shows the case where the cancer cells have been treated with EGF. In this case, the level of *P*_2_ increases but the level of *P*_1_ decreases relative to the other cases. It appears clear that the simulation is capable of showing that glucose flux shunt from glycolysis to either the pentose phosphate pathway or the serine biosynthesis at ensemble levels, whose trend is indeed consistent with the already known functions of the glucose flux regulators in populations^24–29^. Collectively, our mathematical model with high-content imaging analysis is adequate to predict metabolic consequences of glucose flux regulators in a population of cancer cells.

**Table 5:** Ratios of cell distribution showing different-sized clusters in the five environments at the population level.

	No cluster	Small	Medium	Large	Total
Hs578T (Control)	1.6%	58.3%	13.4%	26.7%	100%
Hs578T with Methylene Blue	0.5%	43.0%	25.7%	30.8%	100%
Hs578T with Fructose-1,6-Bisphosphate	0.0%	45.3%	29.1%	25.6%	100%
Hs578T with Epidermal Growth Factor	0.4%	53.1%	7.6%	38.9%	100%
Hs578T with 2-Deoxyglucose	0.0%	34.7%	21.2%	44.1%	100%

### Mathematical Prediction of Metabolic Flux with 2-Deoxyglucose at Ensemble Levels

Our model is now applied to assess metabolic consequences of 2-deoxyglucose in Hs578T cells. 2-Deoxyglucose is a competitive inhibitor of phosphoglucose isomerase catalyzing step 2 in glycolysis^30^. However, due to the lack of 2-hydroxyl group, 2-deoxyglucose cannot further metabolize into fructose-6-phosphate in downstream glycolysis, thereby resulting in the inhibition of glycolysis. Accordingly, 2-deoxyglucose appeared to impair cell growth and thus implemented for anti-tumor therapeutics. However, its pharmacological effects on cancer treatment^31–33^ remain inconclusive, consequently raising a number of questions particularly including potential metabolic alterations by 2-deoxyglucose in cancer cells.

**Figure 7:**
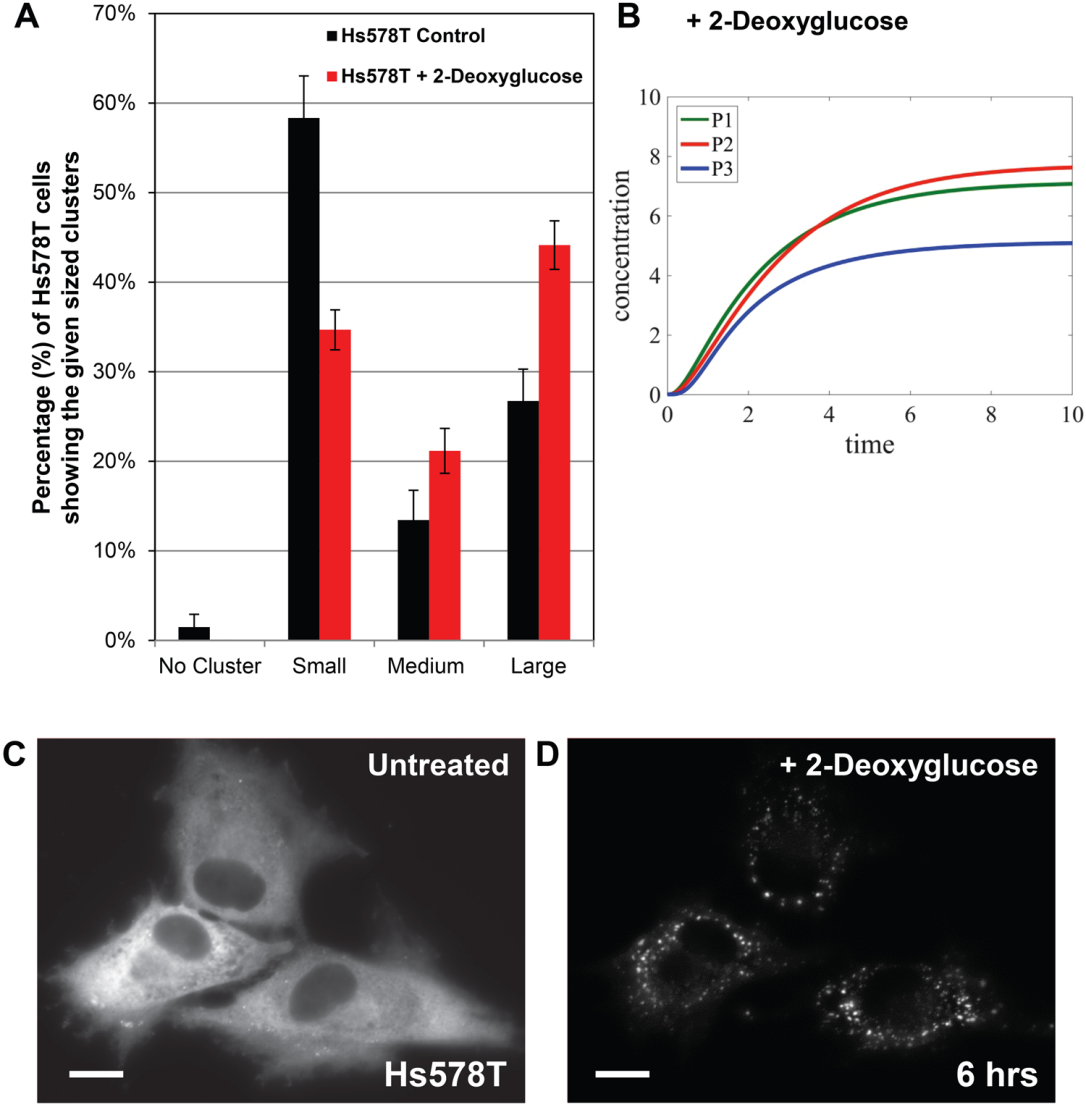
The effect of 2-deoxyglucose on the distribution of Hs578T cells with various sizes of PFKL-mEGFP clusters. The percentage (%) of Hs578T cells displaying each size of PFKL-mEGFP cluster was analyzed in the presence of 2-deoxyglucose. (**A**) The graph shows the average percentages (%) of cells displaying the given sized clusters along with their standard deviations (±) in the absence (black bars) and presence (red bars) of 2-deoxyglucose. At least five independent imaging sessions were performed and total 1200 transfected cells were analyzed. Statistical analyses were performed using two-sample two-tail *t*-tests. *p < 0.01. (**B**) Metabolic flux analysis were performed using our mathematical model with the high-content imaging data of 2-deoxyglucose. Relative to a control flux (Fig. 2A), glycolytic flux (*P*_3_) decreased, but the metabolic shunts of glucose to the pentose phosphate pathway (*P*_1_) and serine biosynthesis (*P*_2_) increased. (**C** and **D**) Representative images of Hs578T cells show subcellular localization of PFKL-mEGFP before and after treatment of 2-deoxyglucose (25 mM) for 6 hours. Scale bar, 10 µm.

In this work, we have employed our mathematical model to predict whether 2-deoxyglucose can potentially alter the direction of glucose flux in cancer cells beyond glycolysis inhibition. First, we performed cell-based high-content imaging assays to validate the metabolic effect of 2-deoxyglucose at single cell levels (Fig. 7). Briefly, we expressed PFKL-mEGFP as a glucosome marker in Hs578T cells. To be consistent with Table 5, Hs578T cells were cultured in RPMI1640 with 10% dialyzed FBS. When we treated transfected Hs578T cells with 25 mM of 2-deoxyglucose for 6 hours, the percentage of cells showing small-sized clusters was significantly reduced from 58.3 % to 34.7% (Fig. 7A). Meanwhile, the percentages of cells showing medium- and large-sized clusters were significantly increased from 13.4% to 21.2% and from 26.7% to 44.1%, respectively (Fig. 7A), indicating the strong promotion of glucosome formation and thus metabolic shunts to anabolic pathways.

Subsequently, we computed an ensemble-level outcome of glucose flux in the presence 2-deoxyglucose. Similarly to Fig. 6, we calculated the changes of metabolic outcomes at ensemble levels based on our subcellular flux analysis (Fig. 2) and high-content imaging analysis (Fig. 7A, Table 5). Relative to a control flux in Hs578T cells where glycolytic flux reaches to ~7 arbitrary units at *t* = 10 (Fig. 6A), glycolytic flux was indeed inhibited at ~ 5 arbitrary units in the presence of 2-deoxyglucose (Fig. 7B). Importantly, our model provided that metabolic shunts from glycolysis to anabolic biosynthetic pathways were promoted and relatively became dominant in the population of Hs578T cells. Although more experimental validation may be necessary, it is clear that 2-deoxyglucose promoted the formation of glucosome clusters in both single-cell (Fig. 7C,D) and ensemble levels (Fig. 7B). Therefore, we propose that along with the inhibitory role of 2-deoxyglucose in glycolysis, its commitment diverting glucose flux into anabolic pathways may explain in part cancer progression observed during its clinical trials^31^.

## Discussion

We constructed a simple mathematical model to understand the cluster size-dependent functional contributions of metabolic enzymes and their multienzyme complexes to cancer cell metabolism. Briefly, glycolysis is interconnected with energy metabolism and anabolic biosynthetic pathways, including the pentose phosphate pathway and serine biosynthesis. Three metabolic products (*P*_*i*_) thus represent downstream pathways of glycolysis, allowing us to investigate how glucose flux changes its direction at metabolic nodes between energy metabolism and anabolic pathways in cancer cells. Based on our experimental data^4^, we developed a model to predict that medium-sized clusters of glucosomes shunt glucose flux into the pentose phosphate pathway whereas large-sized clusters of glucosome divert glucose flux into serine biosynthesis at subcellular levels. Importantly, our model supports that the changes of relative ratios of cancer cells displaying small-, medium- and large-sized clusters in a population appear to be significant enough to influence overall net metabolic outcomes of cancer cells at ensemble levels. Moreover, our mathematical model is further evaluated to predict the effect of 2-deoxyglucose on the fate of glucose, thus providing new quantitative insights of how 2-deoxyglucose alters glucose flux in cancer cells. Collectively, we conclude that our mathematical model supports the hypothesis that glucosomes divert glucose flux into the pentose phosphate pathway and serine biosynthesis in a cluster size-dependent manner.

It is important to emphasize here that our mathematical model accounting for size-dependent metabolic functions of glucosome clusters is significantly different from other models that mostly rely on *in vitro* enzyme kinetics. Our model explains altered glycolysis in cancer cells that redirects glucose flux into the pentose phosphate pathway and serine biosynthesis in a cluster size-dependent manner. Importantly, we can now predict the direction of glucose flux in the presence of small-molecule drug candidates as long as quantitative high-content imaging data is obtained in single-cell levels. Additionally, we can quantify relative partition ratios of glucose flux between glycolysis, the pentose phosphate pathway and serine biosynthesis in various conditions. Collectively, our mathematical model fully integrates various cancer-associated mechanisms discovered in recent years, thus advancing our understanding of cancer cell metabolism in single cells.

## Electronic supplementary material

Supplementary Information
