# Supplementary material for: A Mathematical Model for Enzyme Clustering in Glucose Metabolism
Source: Sci Rep. 2018 Feb 9;8:2696. doi: 10.1038/s41598-018-20348-7 (PMC5807315; doi:10.1038/s41598-018-20348-7)
Supplement: Supplementary file 1 — Supplementary Information [file 41598_2018_20348_MOESM1_ESM.docx]

**Supplementary Information**

**A Mathematical Model for Enzyme Clustering in Glucose Metabolism**

Miji Jeon^1^, Hye-Won Kang^2^*, and Songon An^1^*

^1^Department of Chemistry and Biochemistry, and ^2^Department of Mathematics and Statistics, University of Maryland Baltimore County (UMBC), 1000 Hilltop Circle, Baltimore, MD 21250, USA

*To whom correspondence should be addressed to: Dr. Hye-Won Kang ([hwkang@umbc.edu](mailto:san@umbc.edu)) and Dr. Songon An ([san@umbc.edu](mailto:san@umbc.edu))

1. **Supplemental Figure S1.**
2. **Supplemental MATLAB code 1: Simulation of Glucose Metabolism**
3. **Supplemental MATLAB code 2: Figures 2, 4 and 5.**
4. **Supplemental MATLAB code 3: Figures 6 and 7B**

**
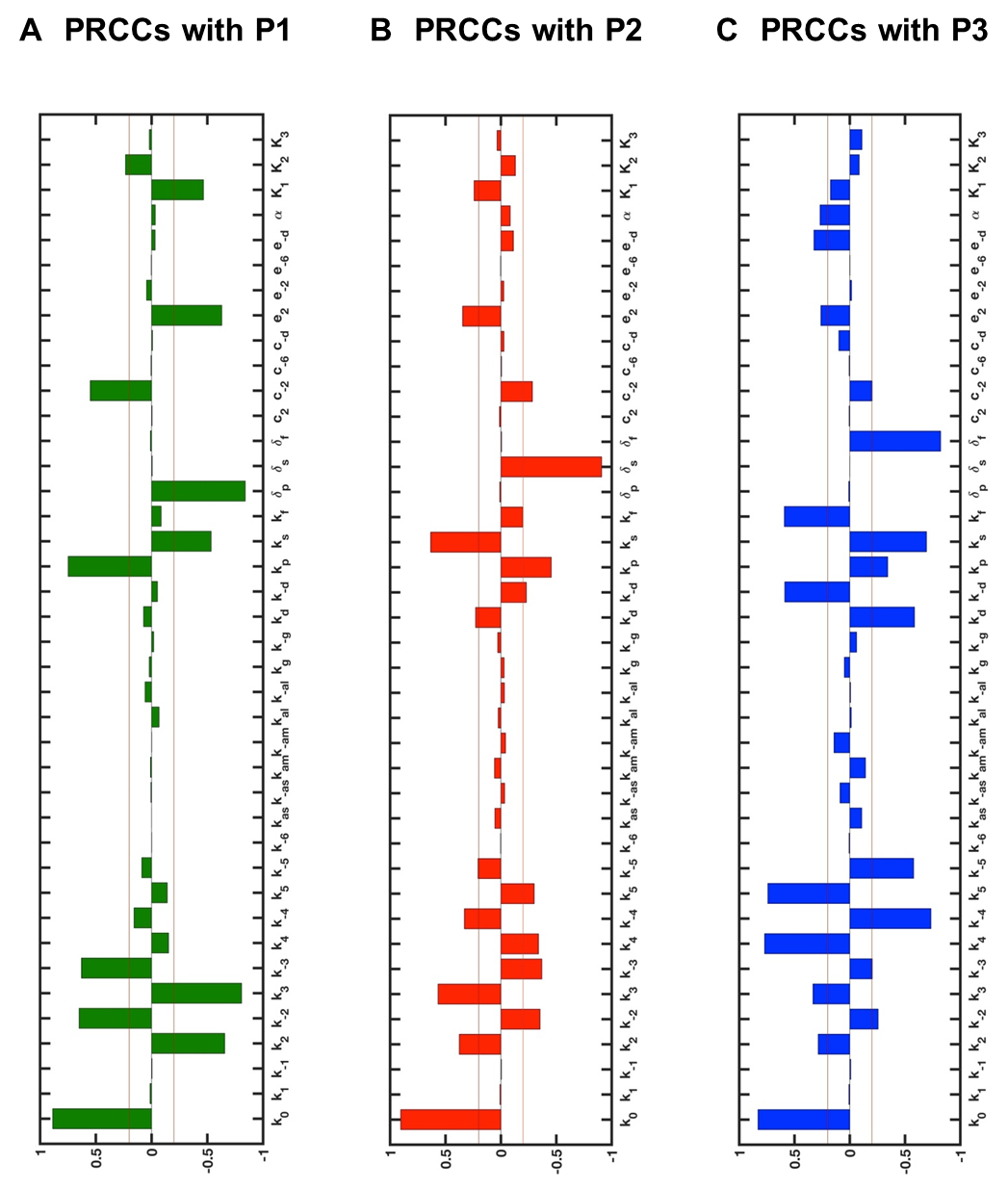
**

**Supplemental Figure S1. The partial rank correlation coefficients (PRCCs) between all the input parameters and the concentrations of metabolic products.** The PRCCs with $P_{1}$, $P_{2}$, and $P_{3}$ , respectively, are graphed to provide relative strengths of the correlations between the input parameters and the concentrations of metabolic products. The lines of +0.2 and -0.2 indicate the thresholds we used to distinguish sensitive essential parameters from non-essential parameters. Figure 3 show the PRCC values of sensitive essential parameters for each product. $P_{1}$, $P_{2}$, and $P_{3}$ represent metabolic outcomes of the pentose phosphate pathway, serine biosynthesis and the downstream of glycolysis, respectively.

**Supplemental MATLAB code 1: Simulation of Glucose Metabolism**

ODEs to simulate glucose metabolism.

function dydt = glucosome_ode(t,y,p)

% assign input parameters

k0=p(1); k1=p(2); k_1=p(3); k2=p(4); k_2=p(5);

k3=p(6); k_3=p(7); k4=p(8); k_4=p(9); k5=p(10);

k_5=p(11); k_6=p(12); kas=p(13); k_as=p(14); kam=p(15);

k_am=p(16); kal=p(17); k_al=p(18); kg=p(19); k_g=p(20);

kd=p(21); k_d=p(22); kp=p(23); ks=p(24); kf=p(25);

delta_p=p(26); delta_s=p(27); delta_f=p(28); c2=p(29); c_2=p(30);

c_6=p(31); c_d=p(32); e2=p(33); e_2=p(34); e_6=p(35);

e_d=p(36); alpha=p(37); K1=p(38); K2=p(39); K3=p(40);

% assign variables

S1=y(1); S2=y(2); S3=y(3); S4=y(4); S5=y(5); S6=y(6); S7=y(7);

E1=y(8); E2=y(9); E3=y(10); E4=y(11); ES=y(12); EM=y(13); EL=y(14);

E3i=y(15); E1gly=y(16); P1=y(17); P2=y(18); P3=y(19);

% ode equations

dydt = [k0 - k1*S1 + k_1*S2;...

%y(1) S1

k1*S1 - k_1*S2 - k2*(E1+ES+c2*EM+e2*EL)*S2*K1/(K1+S3)...

+ k_2*(E2+c_2*EM+e_2*EL)*S3*K2/(K2+S2) - kp*S2;...

%y(2) S2

k2*(E1+ES+c2*EM+e2*EL)*S2*K1/(K1+S3)...

- k_2*(E2+c_2*EM+e_2*EL)*S3*K2/(K2+S2) - k3*S3...

+ k_3*S4;...

%y(3) S3

k3*S3 - k_3*S4 - k4*S4 + k_4*S5 - ks*S4;...

%y(4) S4

k4*S4 - k_4*S5 - k5*E3i*S5 + k_6*(E4+c_6*EM+e_6*EL)*S7;...

%y(5) S5

k5*E3i*S5 - k_5*S6 - kf*S6;...

%y(6) S6

k_5*S6 - k_6*(E4+c_6*EM+e_6*EL)*S7;...

%y(7) S7

- kas*E1 + k_as*ES - kg*E1 + k_g*E1gly;...

%y(8) E1

- kam*ES*E2*E3*E4 + k_am*EM;...

%y(9) E2

- kam*ES*E2*E3*E4 + k_am*EM + kd*E3i...

- k_d*(E3+c_d*EM+e_d*EL)*(1+alpha*S3/(S3+K3));...

%y(10) E3

- kam*ES*E2*E3*E4 + k_am*EM;...

%y(11) E4

kas*E1 - k_as*ES - kam*ES*E2*E3*E4 + k_am*EM;...

%y(12) ES

kam*ES*E2*E3*E4 - k_am*EM - 11*kal*EM^(11) + 11*k_al*EL;...

%y(13) EM

kal*EM^(11) - k_al*EL;...

%y(14) EL

- kd*E3i + k_d*(E3+c_d*EM+e_d*EL)*(1+alpha*S3/(S3+K3));...

%y(15) E3* (=E3i)

kg*E1 - k_g*E1gly;...

%y(16) E1gly

kp*S2 - delta_p*P1;...

%y(17) P1

ks*S4 - delta_s*P2;...

%y(18) P2

kf*S6 - delta_f*P3];

%y(19) P3

**Supplemental MATLAB code 2: Figures 2, 4 and 5**

This code is the code to run 4 subcellular cases with varying parameters.

clear all

% parameters

k0=10; k1=10; k_1=10; k2=40; k_2=7; k3=10; k_3=10; k4=14; k_4=7;

k5=1; k_5=10; k_6=10; kg=1; k_g=1; kd=1; k_d=1;

kp=5; ks=5; kf=5; delta_p=0.5; delta_s=0.5; delta_f=0.5;

alpha=1; K1=1; K2=1; K3=1;

% initial conditions (19)

y0 = [0.01; 0.01; 0.01; 0.01; 0.01; 0.01; 0.01;...

% S1 S2 S3 S4 S5 S6 S7

99.99; 100; 99.99; 100; 0; 0; 0; 0.01; 0.01;...

% E1 E2 E3 E4 ES EM EL E3* E1gly

0.01; 0.01; 0.01];

% P1 P2 P3

% Parameter changes in Four Subcellular Cases:

% No Cluster, Small Cluster, Medium Cluster, Large Cluster

for n=0:3

switch n

case 0

% No Cluster

kas=0; k_as=0; kam=0; k_am=0; kal=0; k_al=0;

c2=0; c_2=0; c_6=0; c_d=0;

e2=0; e_2=0; e_6=0; e_d=0;

case 1

% Small Cluster

kas=10; k_as=10; kam=0; k_am=0; kal=0; k_al=0;

c2=0; c_2=0; c_6=0; c_d=0;

e2=0; e_2=0; e_6=0; e_d=0;

case 2

% Medium Cluster

kas=10; k_as=10; kam=10; k_am=10; kal=0; k_al=0;

c2=0.2; c_2=10; c_6=10; c_d=0.1;

e2=0; e_2=0; e_6=0; e_d=0;

case 3

% Large Cluster

kas=10; k_as=10; kam=10; k_am=10; kal=10; k_al=10;

c2=0.2; c_2=10; c_6=10; c_d=0.1;

e2=2.5; e_2=0.1; e_6=10; e_d=0.05;

end

% parameters (40)

p = [k0,k1,k_1,k2,k_2,k3,k_3,k4,k_4,k5,...

k_5,k_6,kas,k_as,kam,k_am,kal,k_al,kg,k_g,...

kd,k_d,kp,ks,kf,delta_p,delta_s,delta_f,c2,c_2,...

c_6,c_d,e2,e_2,e_6,e_d,alpha,K1,K2,K3];

% times ranges

time_ranges = [0:0.005:10];

% solve ODEs with parameters, initial conditions, time ranges

[T,y] = ode15s(@glucosome_ode,time_ranges,y0,[],p);

P1=y(:,17);

P2=y(:,18);

P3=y(:,19);

pl = [k0,k1,k_1,k2,k_2,k3,k_3,k4,k_4,k5,...

k_5,k_6,kas,k_as,kam,k_am,kal,k_al,kg,k_g,...

kd,k_d,kp,ks,kf,delta_p,delta_s,delta_f,c2,1,...

c_6,c_d,e2,e_2,e_6,e_d,alpha,K1,K2,K3]; % c_2 changed

[Tl,yl] = ode15s(@glucosome_ode,time_ranges,y0,[],pl);

lower_P1=yl(:,17);

lower_P2=yl(:,18);

lower_P3=yl(:,19);

pu = [k0,k1,k_1,k2,k_2,k3,k_3,k4,k_4,k5,...

k_5,k_6,kas,k_as,kam,k_am,kal,k_al,kg,k_g,...

kd,k_d,kp,ks,kf,delta_p,delta_s,delta_f,c2,10,...

c_6,c_d,e2,e_2,e_6,e_d,alpha,K1,K2,K3];

[Tu,yu] = ode15s(@glucosome_ode,time_ranges,y0,[],pu);

upper_P1=yu(:,17);

upper_P2=yu(:,18);

upper_P3=yu(:,19);

% Parameter ranges to change in 'pl' and 'pu'

% k2:10~40, k_2:7~10, k_d:1~10

% c2:0.2~1, c_2:1~10, c_6:1~10, c_d:0.1~1

% e2:1~2.5, e_2:0.1~1, e_6:1~10, e_d:0.05~1

figure (n+1)

hold on

s1=plot(T,y(:,17),'-','LineWidth',4);

set(s1,'Color',[0,0.5,0])

s2=plot(T,y(:,18),'-r','LineWidth',4);

s3=plot(T,y(:,19),'-b','LineWidth',4);

legend([s1 s2 s3],'P1','P2','P3','Location','northwest')

set(gcf,'color','w');

set(gca,'FontSize',25,'FontName','Times');

xlabel('time','FontSize',30,'FontName','Times');

ylabel('concentration','FontSize',30,'FontName','Times');

axis([0 10 0 18])

box on

hold off

% Save the figures and data

print('-djpeg','-r1000',['subcellular' num2str(n)])

save(['subcellular' num2str(n) '.mat'],'T','y','P1','P2','P3')

close all

figure(n+5)

hold on

T2 = [T; flipud(T)];

inBetween1 = [yl(:,17); flipud(yu(:,17))];

inBetween2 = [yl(:,18); flipud(yu(:,18))];

inBetween3 = [yl(:,19); flipud(yu(:,19))];

h1=fill(T2, inBetween1, [0,0.5,0]);

h2=fill(T2, inBetween2, 'r');

h3=fill(T2, inBetween3, 'b');

set(h1,'EdgeColor','none','facealpha',.2)

set(h2,'EdgeColor','none','facealpha',.2)

set(h3,'EdgeColor','none','facealpha',.2)

x1=plot(T,y(:,17),'-','LineWidth',4);

set(x1,'Color',[0,0.5,0])

x2=plot(T,y(:,18),'-r','LineWidth',4);

x3=plot(T,y(:,19),'-b','LineWidth',4);

legend([x1 x2 x3],'P1','P2','P3','Location','northwest')

set(gcf,'color','w');

set(gca,'FontSize',25,'FontName','Times');

axis([0 10 0 18])

box on

hold off

% Save the figures

print('-djpeg','-r1000',['c_2_shade' num2str(n)])

close all

end

**Supplemental MATLAB code 3: Figures 6 and 7B**

This is the code to make predictions at the population level using subcellular simulation data (Figure 2) and experimental data shown in Table 5.

clear all

load('subcellular0.mat')

P1_c0=P1;

P2_c0=P2;

P3_c0=P3;

clear P1 P2 P3

load('subcellular1.mat')

P1_c1=P1;

P2_c1=P2;

P3_c1=P3;

clear P1 P2 P3

load('subcellular2.mat')

P1_c2=P1;

P2_c2=P2;

P3_c2=P3;

clear P1 P2 P3

load('subcellular3.mat')

P1_c3=P1;

P2_c3=P2;

P3_c3=P3;

clear P1 P2 P3

save('simul_data.mat')

clear all

load('simul_data.mat')

for k=1:5

% ratios of cell distribution showing different-sized clusters in the five environments

Ratio = [1.6 58.3 13.4 26.7;...

0.5 43.0 25.7 30.8;...

0.0 45.3 29.1 25.6;...

0.4 53.1 7.6 38.9;...

0 34.7 21.2 44.1];

Ratio=Ratio/100;

figure(1)

hold on

s1=plot(T,Ratio(k,1)*P1_c0+Ratio(k,2)*P1_c1+Ratio(k,3)*P1_c2...

+Ratio(k,4)*P1_c3,'-','LineWidth',4);

set(s1,'Color',[0,0.5,0])

s2=plot(T,Ratio(k,1)*P2_c0+Ratio(k,2)*P2_c1+Ratio(k,3)*P2_c2...

+Ratio(k,4)*P2_c3,'-r','LineWidth',4);

s3=plot(T,Ratio(k,1)*P3_c0+Ratio(k,2)*P3_c1+Ratio(k,3)*P3_c2...

+Ratio(k,4)*P3_c3,'-b','LineWidth',4);

legend('P1','P2','P3','Location','northwest')

set(gcf,'color','w');

set(gca,'FontSize',25,'FontName','Times');

xlabel('time','FontSize',30,'FontName','Times');

ylabel('concentration','FontSize',30,'FontName','Times');

axis([0 10 0 10])

box on

hold off

print('-djpeg','-r1000',['population' num2str(k)])

close all

end
